# Recognizing flawed assumptions in suicide risk assessment research and clinical practice

**DOI:** 10.1017/S0033291721002750

**Published:** 2023-04

**Authors:** M. David Rudd

**Affiliations:** University of Memphis, Memphis, TN 38152, USA

To the Editor:

Simpson, Loh, and Goans (2021) make an excellent point about the recent C-SSRS Screener findings, calling for new approaches in research and clinical practice targeting suicide risk assessment in light of poor predictive value estimates in real-world clinical settings like emergency departments. Their recommendation is bolstered substantially by the large sample sizes of both studies cited (*N* = 18 684 and *N* = 92 643), coupled with 30-day and 365-day after ED-visit suicide outcome data. In addition to the recommendation offered by Simpson et al. (2021), these findings reveal a broader problem for research and clinical practice focused on suicide risk assessment, that is, a potential failure to recognize flawed or unacknowledged underlying assumptions driving the effort in some clinical arenas, particularly healthcare and clinical settings.

The use of psychometrically sound screening tools targeting suicidal thoughts have been almost uniformly recommended as a best practice, regardless of clinical setting and related data on predictive value. Suicide risk screening in healthcare settings is undeniably important, with over 80% of those later dying by suicide having been actively engaged in the healthcare system in the year prior, and 50% within the past month. The largest percentage of those dying by suicide after entering the healthcare system pass through primary care settings and emergency departments (National Alliance for Suicide Prevention: Transforming Health Systems Initiative Work Group, 2018). However, the idea that people are ‘falling through the cracks’ is not entirely accurate, as it assumes existing tools are designed to effectively engage and uniformly assess those at significant risk for suicide. As recent C-SSRS data reveal, there may be a more fundamental problem to consider. We may not need better screening tools. Rather, we may need different tools developed for different clinical settings driven by different underlying assumptions in accordance with available data.

Recent findings using ecological momentary assessment (ECA) suggest that large numbers of those at risk for suicide in healthcare settings may not be unwilling to disclose the nature of their risk, rather there is simply significant variability in suicidal ideation and motivation to die from moment to moment, day to day, and week to week that existing tools are unable to accurately capture the needed information in clinically relevant timeframes or are targeting the wrong variables entirely (e.g. Gratch et al., 2019). It has long been recognized that there are many reasons patients choose not to reveal suicidal thoughts, including stigma, fear of hospitalization, along with bias in retrospective recall, with the end resulting being that up to 75% of those that eventually die by suicide deny suicidal thoughts when screened (Berman, 2018). ECA data suggest that for many, it may not necessarily be a choice, rather the very nature of their suicidal thoughts and motivation to die is remarkably fluid, and existing instruments are not developed to capture such fluctuations (Rudd, 2006). Similarly, motivation to die can intensify within narrow windows of time, with 70% of those making suicide attempts making the final decision to act within 1 h of the attempt (Simon et al., 2021). As a meta-analysis by Franklin et al. (2017) demonstrated, suicidal thoughts were of limited value in predicting death by suicide, falling behind psychiatric hospitalization and previous attempts, with all proving to be relatively weak predictors.

It is also critical to recognize potentially important differences between those thinking about suicide for the first time, those making a single attempt, and those with multiple suicide attempts, with converging data over the last several decades indicating that multiple attempters present a profoundly more complex and severe clinical profile across a broad range of variables (Rudd, 2006). Finally, emerging data on access to method suggest access in and of itself can elevate risk independent of current suicidal thinking (e.g. Milner, Witt, Maheen, and LaMontagne, 2017).

Recent findings suggest that machine learning-based suicide risk algorithms targeting electronic health records will likely help close this gap, offering an approach to screening that may well circumvent many of the problems noted above. However, the new approach called for by Simpson et al. (2021) might benefit from the identification of underlying assumptions that provide an empirical foundation to the development of new clinical tools or analytic frameworks:

1. A disproportionately large percentage of patients in healthcare settings are either unwilling or unable to reveal active suicide thinking and motivation to die when responding to direct questions about suicide.
2. Routine variations in suicidal thinking and motivation to die include significant shifts from moment to moment, day to day, and week to week, i.e. from detailed and specific thoughts coupled with significant wish to die and related preparation behavior to fleeting, non-specific thoughts with no significant wish to die.
3. There are some characteristics of chronic suicidal thinking that do not elevate near-term suicide risk and are not clinically meaningful when not coupled with motivation to die and related preparation behavior.
4. Many suicidal patients acknowledge the emergence and sharp increases in motivation to die in distinctively brief windows of time.
5. Access to method, independent of current suicidal thinking, elevates risk for death by suicide.
6. A critical part of the risk assessment process needs to include an evaluation or appraisal of the individual's ability to self-manage elevated risk. Multiple attempters evidence poor or limited capacity for self-management.

## References

[ref1] Berman, A. L. (2018). Risk factors proximate to suicide and suicide risk assessment in the context of denied suicide ideation. Suicide & Life-Threatening Behavior, 48(3), 340–352. 10.1111/sltb.12351. 28429385

[ref2] Franklin, J. C., Ribeiro, J. D., Fox, K. R., Bentley, K. H., Kleiman, E. M., Huang, X., … Nock, M. K. (2017). Risk factors for suicidal thoughts and behaviors: A meta-analysis of 50 years of research. Psychological Bulletin, 143(2), 187–232. 27841450 10.1037/bul0000084

[ref3] Gratch, I., Choo, T., Galfalvy, H., Keilp, J. G., Itzhaky, L., Mann, J. J., … Stanley, B. (2019). Detecting suicidal thoughts: The power of ecological momentary assessment. Depression and Anxiety, 38, 8–16. https://doi.org./10.1002/da.23043. 10.1002/da.23043 32442349

[ref4] Milner, A., Witt, K., Maheen, H., & LaMontagne, A. D. (2017). Access to means of suicide, occupation, and the risk of suicide: A national study over 12 years of coronial data. BMC Psychiatry, 17:125. 10.1186/s12888-017-1288-0. 28376757 PMC5379531

[ref5] National Action Alliance for Suicide Prevention: Transforming Health Systems Initiative Work Group (2018). Recommended standard care for people with suicide risk: Making health care suicide safe. Washington, DC: Education Development Center, Inc.

[ref6] Rudd, M. D. (2006). Fluid vulnerability theory: A cognitive approach to understanding the process of acute and chronic suicide risk. In T. Ellis (Ed.) Cognition and suicide (pp. 355–368) (ISBN: 978-1-59147-357-2). Washington, DC: American Psychological Association.

[ref7] Simon, G. E., Matarazzo, B. B., Walsh, C. G., Smoller, J. W., Boudreaux, E. D., Yarborough, B. J. H., … Shoenbaum, M. (2021). Reconciling statistical and clinicians’ predictions of suicide risk. Psychiatric Services, 72(5), 555–562. 10.1176/appi.ps.202000214. PMC10296777 33691491

[ref8] Simpson, S. A., Goans, C., Loh, R., Ryall, K., Middleton, M. C. A., & Dalton, A. (2021). Suicidal ideation is insensitive to suicide risk after emergency department discharge: Performance characteristics of the Columbia-suicide severity rating scale screener. Academic Emergency Medicine, 1–9. 10.1111/acem.14198. 33346922

[ref9] Simpson, S. A., Loh, R. M., & Goans, C. R. (2021). New data on suicide risk assessment in the emergency department reveal the need for new approaches in research and clinical practice. Psychological Medicine First View, 1–2. 10.1017/S0033291721001653. 34008485

